# Supplementary material for: Identification of photoperiod-induced specific miRNAs in the adrenal glands of Sunite sheep (Ovis aries)
Source: Front Vet Sci. 2022 Jul 22;9:888207. doi: 10.3389/fvets.2022.888207 (PMC9354845; doi:10.3389/fvets.2022.888207)
Supplement: Supplementary file 1 [file Data_Sheet_1.ZIP › Supplementary file/miRDeep2 file.zip!/miRDeep2 file/Know_Structure/SPLP42A2/oar-mir-152.pdf]

miRBase precursor : oar-mir-152
Total read count : 24724
oar-miR-152 read count : 24724
remaining reads : 0

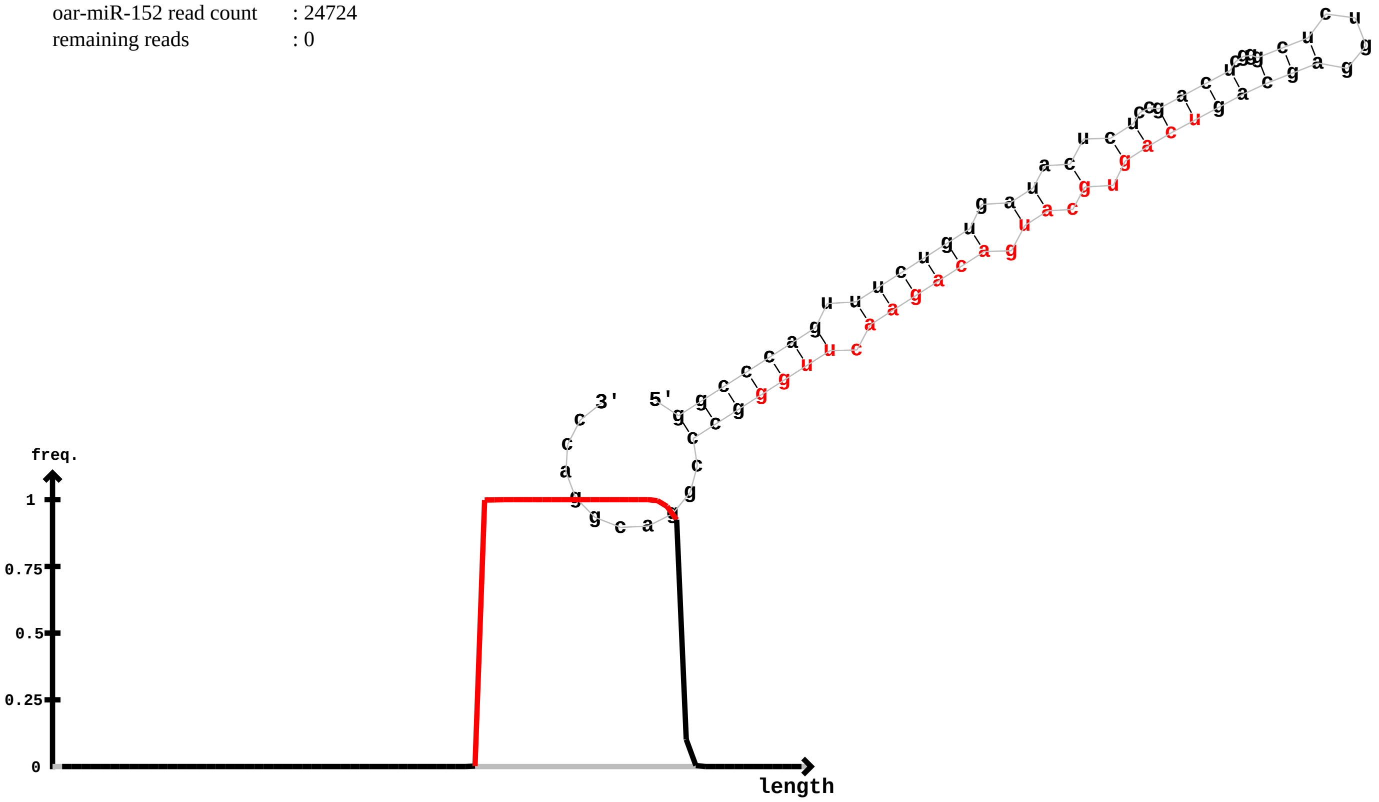

```
                                           oar-miR-152
5'- ggcccaguuucugugauacucuccgacucgggcucuggagcagucagugcaugacagaacuugggcccggacggacc  -3'   exp
    (((((((.((((((.((.(.((..((((...(((....))))))))).).)).)))))).))))))).........   reads   mm   sample
    ..........................................gucagugcaugacagaacuu...............   1       0    seq
    ..........................................gucagugcaugacagaacuug..............   10      0    seq
    ..........................................gucagugcaugacagaacuugg.............   33      0    seq
    ..........................................gucagugcaugacagaacuuggg............   1       0    seq
    ...........................................ucagugcaugacagaac.................   1       0    seq
    ...........................................ucagugcaugacagaacu................   65      0    seq
    ...........................................ucagugcauCacagaacu................   19      1    seq
    ...........................................ucagAgcaugacagaacu................   1       1    seq
    ...........................................ucagugcauCacagaacuu...............   29      1    seq
    ...........................................ucagugcaugacagaacuu...............   509     0    seq
    ...........................................ucagugcaugacagaacuug..............   1220    0    seq
    ...........................................ucagugAaugacagaacuug..............   1       1    seq
    ...........................................ucagugcaugacagaacuugg.............   20302   0    seq
    ...........................................ucagugcaugacagaacuuggg............   2402    0    seq
    ...........................................ucagugcaugacagaacuugggc...........   96      0    seq
    ...........................................ucagugcaugacagaacuugggcc..........   1       0    seq
    ............................................cagugcaugacagaacuu...............   1       0    seq
    ............................................cagugcaugacagaacuugg.............   10      0    seq
    ............................................cagugcaugacagaacuuggg............   2       0    seq
    .............................................agugcaugacagaacuu...............   1       0    seq
    .............................................agugcaugacagaacuug..............   1       0    seq
    .............................................agugcaugacagaacuugg.............   11      0    seq
    .............................................agugcaugacagaacuugA.............   4       1    seq
    .............................................agugcaugacagaacuuggg............   3       0    seq
```
